# The Polymer-Based Technology in the Endovascular Treatment of Abdominal Aortic Aneurysms

**DOI:** 10.3390/polym13081196

**Published:** 2021-04-07

**Authors:** Gianmarco de Donato, Edoardo Pasqui, Claudia Panzano, Brenda Brancaccio, Gaia Grottola, Giuseppe Galzerano, Domenico Benevento, Giancarlo Palasciano

**Affiliations:** Department of Vascular Surgery, University of Siena, 53100 Siena, Italy; pasqui11@student.unisi.it (E.P.); claudia.panzano@gmail.com (C.P.); brenda.brancaccio@gmail.com (B.B.); gaiagrottola90@gmail.com (G.G.); galzerano.giuseppe@gmail.com (G.G.); domenico.benevento@gmail.com (D.B.); palasciano@unisi.it (G.P.)

**Keywords:** vascular surgery, aortic aneurysm, endovascular aneurysm repair, aortic endograft, polyethylene glycol, polymer

## Abstract

An abdominal aortic aneurysm (AAA) is a dilatation of the abdominal aorta that progressively grows until it ruptures. Treatment is typically recommended when the diameter is more than 5 cm. The EVAR (Endovascular aneurysm repair) is a minimally invasive procedure that involves the placement of an expandable stent graft within the aorta to treat aortic disease without operating directly on the aorta. For years, stent grafts’ essential design was based on metallic stent frames to support the fabric. More recently, a polymer-based technology has been proposed as an alternative method to seal AAA. This review underlines the two platforms that are based on a polymer technology: (1) the polymer-filled endobags, also known as Endovascular Aneurysm Sealing (EVAS) with Nellix stent graft; and (2) the O-ring EVAR polymer-based proximal neck sealing device, also known as an Ovation stent graft. Polymer characteristics for this particular aim, clinical applications, and durability results are hereby summarized and commented critically. The technique of inflating endobags filled with polymer to exclude the aneurysmal sac was not successful due to the lack of an adequate proximal fixation. The platform that used polymer to create a circumferential sealing of the aneurysmal neck has proven safe and effective.

## 1. Introduction

### Abdominal Aortic Aneurysm Definition and Treatment Options

An abdominal aortic aneurysm (AAA) is a dilatation in the lower part of the major vessel that supplies blood to the body (aorta). The most accepted definition of AAA is based on a diameter of 3.0 cm or more, which is usually higher than two standard deviations above the mean diameter for men [1,2].

The AAA prevalence and incidence rates have decreased slightly over the last 20 years due to better risk factor management (i.e., smoking cessation, hypertension and diabetes treatment). In 1990, the global prevalence in 75–79-year-olds was 2423 per 100,000 population versus 2275 in 2010. Nevertheless, AAA’s natural history is a progressive growth until it ruptures, leading to life-threatening bleeding [3,4].

Depending on the aneurysm’s size and how fast it is growing, treatment varies from watchful waiting to emergency surgery. To decrease AAA rupture incidence, treatment is typically recommended when the diameter is more than 5 cm. Over the last decades, the treatment options have changed. The traditional approach is represented by the open surgical repair—a longitudinal abdominal incision is performed; abdominal organs are eviscerated and aneurysmectomy is performed with subsequent aortic reconstruction. [5,6] In addition to this approach, a less invasive endovascular treatment has been proposed. Over the years, endovascular aneurysm repair (EVAR) has become the first treatment of choice in patients with suitable anatomy [7,8,9]. EVAR represents a minimally invasive technique that has overcome some critical issues of open surgical repair such as higher intra- and peri-operative risk, the necessity of general anesthesia, intensive Care Unit stay, higher cardiac, pulmonary and renal complications. [10] These advantages led to a constant increasing in the AAA treatment feasibility especially in elderly patients with a substantial number of comorbidities which could be treated with reasonable perioperative risks [11,12] and good early and mid-term outcomes [13,14] also in emergency settings [15].

Thirty years ago, Juan Parodi developed the first prototype of endograft for EVAR [16], a handmade device made of a tube-shaped aorto–aortic graft sutured at each end to a balloon-expandable stent based on the design of radiologist Julio Palmaz. This device was implanted in a human body for the first time on September 7, 1990 in Buenos Aires, Argentina [17]. By 1994, the first commercially available devices had been launched onto the market [18]. Stent-graft material and design changed in various ways to improve conformability, reduce fracture, and minimize device migration rates [19]. 

For years, the basic design was based on metallic stent frames to support the fabric [20]. More recently, a polymer-based technology has been proposed as an alternative method to seal AAA [21,22]. This review underlines the main features of the two platforms that are based on polymer technology. To the best of our knowledge, the literature lacks papers focused on comparing devices that involve polymer-based technology for the endovascular repair of AAA. From this perspective, polymer characteristics, clinical applications, and durability results are summarized and commented critically.

## 2. Polymer and Endovascular Aneurysm Repair

### 2.1. Concept of Traditional Endovascular Aneurysm Repair

**Figure 1:**
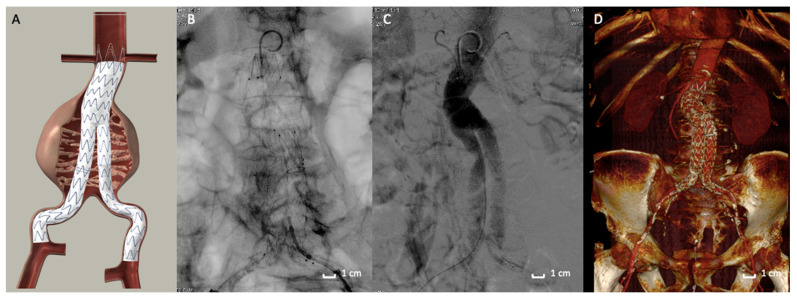
Traditional stent-graft design. (**A**) Picture of the endograft deployed in Abdominal Aortic Aneurysm. Traditional Endovascular aneurysm repair (EVAR) devices employ self-expanding stent structures to promote graft-to-aortic wall apposition (seal) and fixation. (**B**) Intraoperative fluoroscopic image of the endograft deployed in the abdominal aortic aneurysm (AAA). (**C**) Angiographic acquisition that reveals a good exclusion of the AAA and the good patency of the endograft. (**D**) 3D volume rendering of Computed Tomography Angiography at 1-month follow-up that shows the correct aneurysm exclusion.

EVAR has gained wide acceptance as the preferred method to treat patients with AAA [1,23,24,25,26,27,28]. The standard endovascular procedure involves a stent-graft that is designed to exclude the aneurysm from the systemic circulation (Figure 1). The stent is made of a metallic skeleton and covered with polytetrafluoroethylene (PTFE) or polyester fabric that keeps the stent impermeable. The device is advanced through the femoral artery using fluoroscopic guidance toward the aneurysm site and then deployed. Sealing the proximal and distal ends of the aneurysm isolates the aneurysm, preventing subsequent rupture. Traditional EVAR devices employ self-expanding stent structures to promote graft-to-aortic wall apposition (seal) and fixation [29,30,31]. The amount of proximal device oversizing with self-expanding stent grafts (SESGs) is known to influences neck progression over time [32]. As a matter of fact, once deployed, SESGs continue to expand until the nominal diameter is reached unless tissue resistance limits expansion. When aortic neck dilation occurs, midterm outcomes are reportedly adverse [33,34].

Moreover, the “Achilles’ heel” of EVAR is Endoleaks (EL), which consists of persistent blood flow within the aneurysm sac. An EL is a relatively common complication of EVAR, and it could be detected intra-operatively (seen on the on-table angiogram after stent deployment), and during the follow-up [35,36]. Type I EL results from a failing proximal or distal sealing of the aneurysm by the stent graft. Although rare (1–5%), it is a potentially severe complication due to ahigh-flow leakage pressurizing the sac. This condition deserves further treatment to prevent an aneurysm rupture [37,38]. Type II EL is commonly detected during follow-up in 10–25% of EVAR patients [39,40]. It occurs from retrograde low-pressure collateral blood flow into the aneurysm sac, typically from a lumbar artery or the inferior mesenteric artery. Type II ELs can potentially enlarge and pressurize the aneurysm sac until it ruptures, although this rarely happens [41,42].

Challenging anatomies, vessel calcification and thrombosis, iliac vessel tortuosity and complex aortic neck have represented during time some important issues that technological development and research have faced, in order to increase the feasibility and durability of EVAR [43,44]. Ultimately, neck enlargement and type I and II EL are the drawbacks of traditional EVAR devices, which have justified the search for different technologies to seal the aneurysms based on polymers’ use [45,46,47,48].

### 2.2. Polyethylene Glycol: A New Tool for EVAR

**Figure 2:**
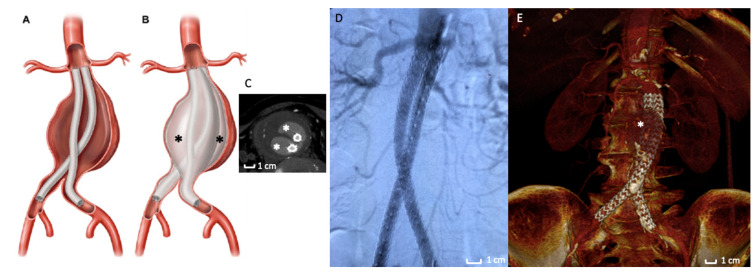
Nellix stent graft. (**A**,**B**) Picture of Nellix endograft during the endobags’ polymer filling maneuvers. The asterisk (*) indicates the endobags that adapt to the morphology of the entire aneurysm sac. (**C**) Axial Computed Tomography Angiography detail of a Nellix endograft implanted. The stent grafts (arrows) are located in the middle of endobags, which are filled with polymer (*). (**D**) Intraoperative image of the Nellix stent graft deployed with angiographic contrast medium that highlights the good patency of the endograft and the exclusion of AAA. (**E**) 3D volume rendering of Computed Tomography Angiography at 3-months follow-up post-implantation that reveals a good aneurysm exclusion. The asterisk (*) indicate the polymer-filled endobags.

**Figure 3:**
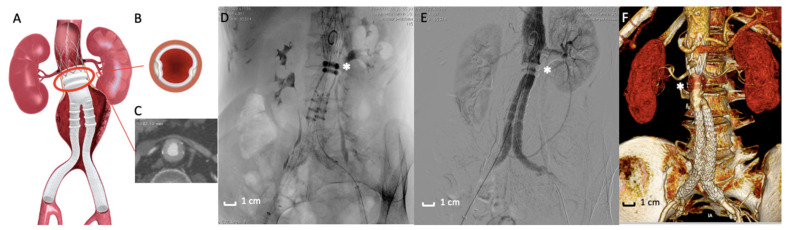
Ovation stent graft. (**A**) Picture of the Ovation endograft. Proximal sealing is assured by polymer filled O-rings (red circle) adapted to the aortic neck anatomy. (**B**,**C**) Details of the axial appearance of O-rings well-opposed to the aortic wall. (**D**) Intraoperative image of the Ovation stent graft deployed. The asterisk (*) highlights the polymer filled O-rings. (**E**) Angiographic acquisition that reveals the good exclusion of the AAA by the polymer filled O-rings (*). (**F**) 3D volume rendering of Computed Tomography Angiography at 3-months follow-up that shows the correct exclusion of the AAA. The asterisk (*) indicates the polymer filled O-rings located in the proximal fixation zone.

The use of polymer as an alternative to traditional SESGs is a fascinating field in endovascular techniques. Two different stent graft designs combined with a polymer technology have been proposed for clinical application in the treatment of AAAs: (1) the polymer-filled endobags, so-called Endovascular Aneurysm Sealing (EVAS) with Nellix stent graft (Figure 2); and (2) the O-ring EVAR polymer-based proximal neck sealing device, so-called Ovation stent graft (Figure 3).

The polymer used in both platforms is the Polyethylene Glycol (PEG)-base one. PEG is a synthetic, non-biodegradable polymer widely used in the biomedical field, especially in tissue engineering research [49,50,51]. PEG is highly biocompatible but also biochemically inert and well suited for biological uses. Moreover, its non-immunogenicity and resistance to protein adsorption increase its applications [52].

PEG-based polymers have a long history of use in medical products [53]. Before approval, the polymer for EVAR passed the rigorous biocompatibility testing required by the international standard on biocompatibility (ISO 10993-1), and these results were reviewed to receive Conformité Européenne (CE) Mark and Food and Drug Administration (FDA) approval. In 2014, the 14-min CustomSeal Fill Polymer Kit, which replaced the Ovation Fill Polymer Kit, received both CE and FDA approval.

A significant amount of nonclinical testing was performed to evaluate the polymer kit [54]:

- Biocompatibility: comprehensive biocompatibility testing was performed in accordance with the EN ISO 10993-1:2009/AC:2010 standard to evaluate the safety of the materials, including toxicity, sensitization, hemocompatibility, and irritation/intracutaneous reactivity (13-week intramuscular implant). All test results confirmed products are biocompatible and safe for implanting in humans.
- Bench testing: extensive engineering testing was performed on the bench to evaluate the design characteristics in accordance with the EN ISO 25539-1:2009 cardiovascular implants—endovascular devices—part 1: endovascular prostheses. All test results confirmed the products met their design specifications.
- Animal testing: chronic animal studies showed no evidence of structural changes in the fill polymer. A chronic animal study demonstrated no adverse effects associated with deliberate release of the fill polymer within the animals’ vasculature. In particular, no evidence of embolization, toxic response, or end-organ abnormality at 30 days was recorded.

These results were reviewed to receive CE Mark and FDA approval.

Over time, polymer has been used in two techniques: the first one uses the polymer as a filling material for the aneurysm sac (EVAS) (Figure 2A–C,E), [55] while the second uses the polymer as a system for sealing and stabilizing the prosthesis at the level of the proximal aneurysmal neck (Ovation Endograft) (Figure 3A–F) [56,57]. (Table 1) The following paragraphs underline the clinical applications and the two stent graft designs’ durability results with a polymer-based technology for AAA treatment.

### 2.3. The Polymer-Filled Endobags, Endovascular Aneurysm Sealing (EVAS) with Nellix Stent Graft

The Nellix System (Endologix Inc., Irvine, CA, USA) for endovascular aneurysm sealing (EVAS) was introduced as a novel approach to the treatment of AAA [58] in 2013. The EVAS procedure is conceptually distinct from EVAR: the Nellix device is designed to seal and obliterate the aneurysm lumen and not to exclude it (Figure 2B,C).

The device consists of two balloon-expandable stents supporting the aorta flow channel. The system is inserted into the aorta in a similar way to EVAR. By using guidewires, the system is advanced into the aorta through the femoral arteries. The catheter sheaths are then pulled back, deploying the device, which expands from the non-aneurysmal aorta proximally to the iliac arteries distally. The non-porous PTFE-based endobags are then filled with the biocompatible polyethylene glycol polymer (Figure 2B,C), which adjusts the endobag to fit the aneurysm sac lumen. The bio-stable cross-linked polymer inflating the endograft envelope in opposition to the aortic wall mimics the process of injection molding. Thus, high conformability is achieved, as the device is molded in-situ to the specific patient anatomy to accomplish a customized seal. Once this configuration is completed, the polymer cross-links to form a ‘personalized’ prosthesis [59].

Following CE marking in 2013, early reports suggested that EVAS may allow the treatment of complex aneurysms repair and may prevent type II endoleak formation [60]. In the first clinical reports by selected centers in a controlled setting of correct clinical indications and appropriate device implantation, [61,62] the 30-day and 1-year outcomes of EVAS using the Nellix device were encouraging, and safety in the short term appeared to be established [63].

However, after more extensive use by several centers with more liberal indications regarding the use of the device in more challenging anatomical situations, the safety and durability of the procedure with Nellix became very controversial. Lately, more extended follow-up data showed a high rate of graft failure at two years and beyond [64]. In 2016, the EVAS FORWARD, an investigational device exemption trial, reported higher than expected rates of distal migration (6% incidence in a dedicated root-cause analysis), type IA endoleak, and aneurysm sac expansion, leading to a refinement of the instructions for use (IFU) [65]. The new IFU suggested that EVAS would be most effective and durable in large AAA with minor thrombus and neck criteria similar to the IFU for another commercially available EVAR stent grafts.

**Figure 4:**
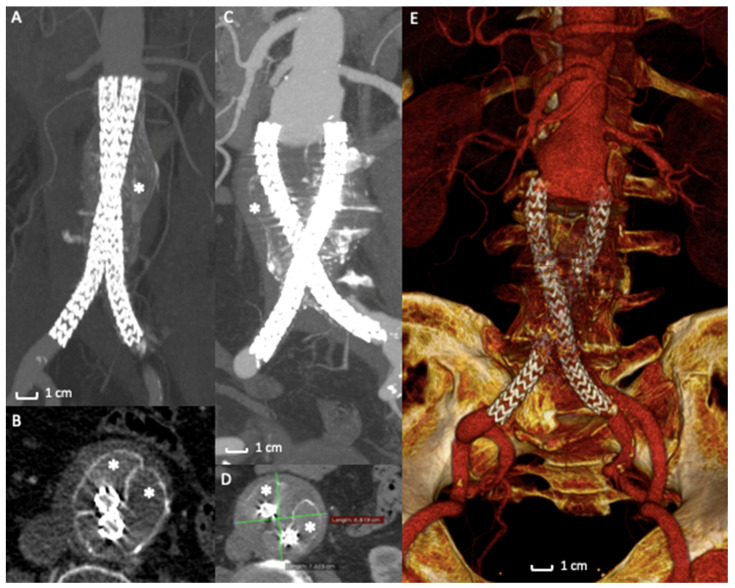
Case of Nellix Endograft Failure due to distal migration. (**A**) Computed Tomography Angiography detail of a Nellix endograft implantation. The asterisk (*) indicates the polymer filled endobags that correctly seal the aneurysm sac. (**B**) Axial view of the same exam highlights the correct stents’ and endobags’ configuration, asterisks (*) mark the two endobags adapted to the aneurysm sac. (**C**) Computed Tomography Angiography detail of the 2-year follow-up exam revealing a distal migration of the entire system (arrows) with AAA enlargement (**D**) and endobags dislocation (*). (**E**) 3D volume rendering of Computed Tomography Angiography of the endograft migration and failure, requiring open surgical conversion.

In a retrospective analysis [66] of prospectively collected data of 295 cases of endovascular aneurysm sealing using the Nellix device for abdominal aortic aneurysm, therapeutic failure was seen at a surprising and alarming rate of 33.2%. The most common failure mechanism was the stent graft migration associated with type IA endoleak and sac expansion, as demonstrated by a recent systematic review (Figure 4A–E) [58]. In several cases of failure, stent grafts were explanted from the patients’ bodies with a significant open vascular intervention. EVAS failures were also responsible for AAA rupture with severe or fatal consequences for the patients. In case of graft explantation, surgeons noted that the bags containing polymer were still intact, the polymer was not leaking into the vasculature, and maintained the expected consistency. The cause of failure was reported to be the lack of a stable proximal fixation of the entire device into the aneurysm sac [67,68,69].

Following the above-mentioned negative feedback on EVAS procedures’ durability, the manufacturer voluntarily recalls the device, and the CE mark was suspended in 2019 [70].

In conclusion, a polymer filling the aneurysmal sac does not ensure the exclusion of the aneurysm because of the lack of proximal fixation. The real problem was in designing the stent grafts without proximal hooks or barbs to guarantee adequate fixation, rather than a failure of polymer technology by itself.

### 2.4. The O-Ring EVAR Polymer-Based Proximal Neck Sealing Device, So-Called Ovation Stent Graft

The first idea to use a polymer to seal the aortic neck by an endovascular operation was reported in 2007. The device with such technology was called Enovus (Trivascular, Boston Scientific, Santa Rosa, CA, USA). It consisted of polymer-filled channels located along the entire PTFE fabric of the endograft body. The endograft ring-shaped cavity was filled with three radiopaque, epoxy components that solidify, creating a rigid scaffold. This device was the first that used polymer with the distinct feature of substituting the endograft metallic stents, allowing radial and longitudinal support. The device was tested on an animal model [71] but did not receive the approval for clinical use on humans. Unfortunately, the clinical trials revealed some cases of fracture of the suprarenal stent. The load created by anatomical flexing seemed concentrated across a small number of points, leading to a higher potential for fatigue. This problem was rapidly solved with a new design of more uniform strut width which spread g stress-strain loads more evenly across the stent, thereby significantly improving the resistance to fatigue.

A few years later, an evolution of the Enovus was proposed for human application with the name of Ovation Endograft (Endologix, Irvine, CA, USA). After successful animal investigations [72], the platform was tested in clinical trials with positive feedback, which was a fundamental evolution in the use of polymers in the EVAR field. The endograft is based on a new concept design separating fixation from sealing—fixation activity is assured by a suprarenal stent and anchors (not different from traditional suprarenal EVAR devices), while sealing activity is based on inflatable O-rings filled with a low-viscosity, non-embolic, radiopaque fill polymer instead of traditional self-expanding stent grafts. The adaptability of the proximal O-ring filled with polymer to the patients’ aortic neck anatomy determines a substantial increase in EVAR feasibility [73,74]. In other words, polymer injected in the O-rings creates a customized circumferential sealing at the proximal aneurysmal neck level, without executing any outward pressure on the aortic neck wall once the final deployment is completed. This assumption comes with a benefit in reducing aortic neck dilatation and consecutive endograft migration, a typical complication of SESGs. All these features define the revolutionary concept of “Custom Seal” as a tailored adaptation of polymer rings to the aortic wall, including wall calcification, wall thrombosis, and aortic tortuosity [75,76,77].

Based on polymer channels rather than traditional metallic stent frames supporting the fabric, the platform has a lower profile of delivery sheath, which is essential to bring the endograft from the groin to the abdominal aorta. The Ovation stent graft has a 14 Fr (4.66 mm) profile that is significantly lower than the 18–20 Fr (6–6.66 mm) profile of traditional SESGs. With such a low-profile delivery catheter, approximately 90% of men and 70% of women with AAA have access vessel diameters considered fit for endovascular repair [78,79,80].

In the beginning, the innovation of using a polymer at the level of the proximal neck raised some worries regarding the correct sealing of the aortic neck over time, the possible aortic neck evolution, stability and durability of polymer, and the possibility of polymer leakage with a consecutive harmful complication for the patients [81].

All these concerns disappeared when data from single-center experiences [82,83] were reinforced by data of large patient population cohort treated with this kind of polymer technology. The ENCORE database enrolled more than 1200 patients undergoing EVAR with Ovation endograft with 50% of cases defined as complex anatomy (neck length <10 mm, neck diameter >28 mm, neck angle >60°, reverse neck taper >10%, distal common iliac artery diameter <10 mm, or external iliac artery diameter <6 mm). Technical success was reached in 99.7%, highlighting the ability of the endograft to conform to difficult anatomies [84], data subsequently confirmed by several studies [74,85,86].

The importance of polymer in the proximal endograft zone is even greater in the exploratory experience of the use of the Ovation endograft in the treatment of patients with Juxtarenal-AAA (those aneurysms involving the infrarenal abdominal aorta adjacent to or including the lower margin of renal artery origins [87]) and is unfit for traditional open surgery repair of Fenestrated-EVAR. In these particular cases, the endograft is deployed, letting the sealing ring land very close to the lowermost renal artery (Figure 3A,E,F) [88,89]. The correct deployment assures the stability of this implant of the endograft and, in particular, by the sealing activity of polymer rings, including the O-ring capability to adapt to irregular anatomies.

The durability of the O-ring EVAR polymer-based devices is due also to the minimum oversize (10–15%) at the proximal seal zone, as opposed to traditional self-expanding stents, which generally require to be oversized by 20% to 30% to ensure apposition of the stent graft to the aortic wall. This rate of oversizing leads to continuous outward radial force being applied to the aneurysm neck [90,91,92]. A meta-analysis of neck dilation after EVAR, showed patients with neck dilation were more likely to develop type IA endoleak (persistent filling of the AAA due to incomplete seal or ineffective seal at the proximal end of the stent graft), experience graft migration, or undergo reintervention [93].

In a recent analysis of patients treated with the O-ring EVAR polymer-based proximal neck sealing platform, at a minimum follow-up CT scan of 24 months, neither aortic neck dilation nor stent graft migration occurred [32]. This finding may suggest that aortic neck evolution could not be associated with EVAR at midterm follow-up when an endograft with no chronic outward radial force is implanted.

As a result, from the Ovation stent graft’s innovative design and polymer use, a new version called Ovation Alto has become available to the market. The conformable O-rings still characterize the new stent graft with CustomSeal™ polymer repositioned near the top of the endograft, providing a seal just below the renal arteries, allowing the treatment of even more complex aortic anatomies. Early experiences highlighted 100% of technical success, no type I/III endoleak, stent-graft migration, abdominal aortic aneurysm rupture, abdominal aortic aneurysm-related mortality, or secondary intervention at 12-months follow-up [94]. The potential dark side of this polymer-based endograft is the polymer leakage outside the fill channels. The polymer system exposure has been reported as a rare complication, responsible for hypersensitivity reaction and anaphylactic shock [95,96,97].

While the unique sealing mechanism of the Ovation device allows a lower profile and improved sealing ability, patient safety was a primary driver during the stent graft development. As part of the process for approval by regulatory agencies worldwide, the Ovation System’s polyethylene glycol (PEG)–based polymer was chosen because it is non-thrombogenic [98] and biocompatible [99,100]; it was designed to be soluble and non-embolic if spilled accidentally into the vasculature after mixing.

In early 2012, a caution statement was approved for the Ovation fill polymer and included in the Instructions for Use (IFU); it warned against balloon dilation of the sealing rings within 20 min of mixing the polymer to avoid potential damage to or compromise of the sealing rings. At the same time, the IFU were also updated to include instructions to manage patients who experience a hypersensitivity reaction to the device materials in accordance with standard recommendations for treatment of patients with radiocontrast allergies (e.g., antihistamines, corticosteroids, and adrenaline) [101].

Gupta N. and colleagues reviewed all reports related to polymer leaks with the device received by the manufacturer. The polymer leak was reported in 26 patients over more than 10.000 endograft implants. Factors that seem to contribute to the risk of polymer leak include the initial manufacturing process for the device, excessive graft manipulation, early ballooning before complete polymer cure and lower body temperature, which can slow the polymer cure rate [102].

## 3. Conclusion and Future Perspectives

Polymer-based technology has been proposed as alternative methods for treating AAA with an endovascular approach. Two different polymer-based platforms have been developed for clinical purposes. The technique of inflating endobags filled with polymer combined with stents to exclude the aneurysmal sac was not successful due to the lack of an adequate proximal fixation of the device. The polymer per se was not responsible for the failure of this platform. The manufacturer recalled the device from the market. Some prototypes are now being tested for the efficacy of endobags filled with polymer combined with stents equipped with barbs for adequate proximal fixation. The device that used polymer to create a circumferential sealing of the aneurysmal has proven safe and effective. However, some rare cases of polymer spill over into the human vasculature may generate systemic reactions requiring careful clinical monitoring and treatment. By adapting the O-ring to the aortic wall, this polymer-based platform offers a customized sealing of the aneurysm, which remain stable over the years without influencing neck dilatation. Finally, the low profile of such a platform based on polymer channels rather than traditional metallic stent frames to support the fabric may expand patient applicability for a minimally invasive EVAR treatment. The impact of this kind of technology is already substantial. The possibility of using polymer characteristic to create a customized sealing of the aneurysm have been a great revolution in EVAR procedures. The unification of the main features of both endografts could become the main research direction of the future endograft development. It could lead to a significant evolution of EVAR, allowing the creation of a novel device characterized by a tailored proximal sealing obtained from the Ovation endograft and complete obliteration of the residual aneurysmal sac taken from the Nellix Endobags technology. This combination could determine a significant double effect—reducing the incidence of Type I (leak at proximal graft end for inadequate seal) and II Endoleak (aneurysmal sac filling via branch vessel) that represents two of the most important critical issues of the endovascular management of the aortic aneurysmal disease. Further application of polymer-based technology is expected in the following year to treat other vascular diseases such as thoracic and thoracoabdominal aortic aneurysms and also thoracic dissections in an urgent/emergent setting.

## Figures and Tables

**Table 1:** Main characteristics of the two polymer-based platforms used in the Endovascular Aneurysm Repair Procedures.

Type of Endograft	Type of Polymer Used	Type of Endograft Fixation	Polymer Location	Main Advantages	Eventual Critical Issues
Ovation stent graft	PEG	Proximal	O-rings network	-Reduction of endograft profile -No aortic neck stress due to endograft radial force	-Polymer leakage -Stability and durability of proximal endograft fixation
Nellix stent graft	PEG	Aneurysm sac obliteration	Endobags	-Aneurysmal sac complete obliteration -Prevention of type II EL	-Lack of proximal fixation -Polymer leakage

* PEG: Polyethylene-glycol; EL: Endoleak.

## Data Availability

No new data were created or analyzed in this study. Data sharing is not applicable to this article.

## Abbreviations

EVAR: Endovascular Aneurysm Repair; AAA: Abdominal Aortic Aneurysm; PTFE: polytetrafluoroethylene; SESGs: self-expanding stent grafts; PEG: Polyethylene Glycol; EL: Endoleak; EVAS: Endovascular Aneurysm Sealing; IFU: Instructions for Use; CE: Conformité Européenne.
